# Efficacy and safety of acupuncture for dysphagia after radiotherapy or chemoradiotherapy in patients with nasopharyngeal carcinoma: a systematic review and meta-analysis

**DOI:** 10.3389/fonc.2026.1919320

**Published:** 2026-09-24

**Authors:** Zhanying Lin, Hongliang Shi, Yihao Tao, Hanran Liu, Liren Chen, Jinduo Zhao, Shuang Liu

**Affiliations:** 1 The Second Affiliated Hospital of Tianjin University of Traditional Chinese Medicine, Tianjin, China; 2 Department of Orthopedics, Union Hospital, Tongji Medical College, Huazhong University of Science and Technology, Wuhan, China

**Keywords:** acupuncture, chemoradiotherapy, dysphagia, meta-analysis, nasopharyngeal carcinoma

## Abstract

**Background:**

Acupuncture has been used as an adjunctive supportive intervention for various cancer-related symptoms. However, its efficacy and safety for dysphagia after radiotherapy or chemoradiotherapy in patients with nasopharyngeal carcinoma have not been systematically evaluated.

**Objective:**

To evaluate the efficacy and safety of acupuncture for dysphagia after radiotherapy or chemoradiotherapy in patients with nasopharyngeal carcinoma. The outcomes were post-treatment Water Swallowing Test (WST) scores, the proportion of patients achieving a favorable swallowing status, the clinical effective rate, videofluoroscopic swallowing study (VFSS) outcomes, and Standardized Swallowing Assessment (SSA) scores.

**Methods:**

PubMed, Embase, Web of Science, the Cochrane Library, China National Knowledge Infrastructure, Chinese Biomedical Literature Database, Wanfang Database, and VIP Database were searched from inception to March 2026. Eligible randomized controlled trials were included. Two reviewers independently screened studies, extracted data, and assessed risk of bias using the Cochrane risk-of-bias tool. Meta-analyses were conducted using R software.

**Results:**

Twelve randomized controlled trials including 863 patients were analyzed. Acupuncture reduced post-treatment WST scores compared with control interventions (MD = -0.78, 95% CI: -1.04 to -0.51). The clinical effective rate was also higher in the acupuncture group (RR = 1.17, 95% CI: 1.08 to 1.27). Moreover, acupuncture improved VFSS outcomes (SMD = 0.76, 95% CI: 0.46 to 1.05), reduced SSA scores (MD = -7.65, 95% CI: -12.30 to -3.01), and improved quality of life and nutritional indicators. Safety reporting was limited, precluding definitive conclusions regarding the safety of acupuncture in this population.

**Conclusion:**

Acupuncture may have a potential adjunctive role in improving swallowing function, swallowing-related quality of life, and nutritional indicators in patients with dysphagia after radiotherapy or chemoradiotherapy for nasopharyngeal carcinoma. However, the evidence remains limited by small sample sizes, methodological limitations, and heterogeneity across outcomes. Larger, well-designed randomized controlled trials are needed to confirm these findings and further clarify the clinical role of acupuncture.

**Systematic review registration:**

https://www.crd.york.ac.uk/PROSPERO/, identifier CRD420261371533.

## Introduction

Dysphagia is a common and clinically important complication after radiotherapy or chemoradiotherapy in patients with nasopharyngeal carcinoma (1, 2). It may present as difficulty eating or drinking, coughing or choking with liquids, reduced swallowing efficiency, pharyngeal residue, or aspiration (3, 4). These manifestations may arise from radiation-induced mucosal injury, edema, soft-tissue fibrosis, impaired sensorimotor function, and reduced coordination of the swallowing musculature (4, 5, 8). Persistent dysphagia can limit oral intake, increase the risk of malnutrition, dehydration, and aspiration pneumonia, lead to tube-feeding dependence, and markedly reduce quality of life (2, 4, 8).

Although the importance of improving swallowing dysfunction after radiotherapy or chemoradiotherapy for nasopharyngeal carcinoma has been recognized, effective treatment options remain limited. Current management mainly includes swallowing training, physical therapy, dietary adjustment, and nutritional support (6, 7). Radiation-induced fibrosis and sensorimotor dysfunction may limit functional recovery, and some patients continue to experience dysphagia despite rehabilitation (8, 9). Currently, acupuncture has been explored as an adjunctive therapy for swallowing rehabilitation (10), as it may provide sensory stimulation and influence the motor functions involved in the swallowing process. However, it remains uncertain whether it provides a clinically meaningful additional benefit.

Although several clinical studies have evaluated acupuncture in this setting, most were small and methodologically limited, offering only limited evidence (12–23). A systematic synthesis of randomized controlled trial evidence is therefore warranted. Accordingly, this study aimed to evaluate the efficacy and safety of acupuncture for dysphagia after radiotherapy or chemoradiotherapy in patients with nasopharyngeal carcinoma.

## Materials and methods

### Protocol and registration

This review was reported in accordance with the Preferred Reporting Items for Systematic Reviews and Meta-Analyses (PRISMA) 2020 statement. The protocol was registered with the International Prospective Register of Systematic Reviews (PROSPERO; registration number: CRD420261371533).

### Information sources and search strategy

A systematic search was conducted in English-language and Chinese-language databases, including PubMed, Embase, Web of Science, the Cochrane Library, China National Knowledge Infrastructure (CNKI), Wanfang Database, VIP Database, and the Chinese Biomedical Literature Database (CBM). The search covered all records from database inception to March 10, 2026. Search strategies combined database-specific subject headings, where available, with free-text terms related to nasopharyngeal carcinoma, radiotherapy or chemoradiotherapy, dysphagia, acupuncture. Strategies were adapted for each database (**Supplementary Table S1**). To identify additional eligible studies, the reference lists of included studies and relevant reviews were also screened.

### Inclusion criteria

1. Study design: randomized controlled trials.
2. Participants: patients with pathologically or clinically confirmed nasopharyngeal carcinoma who developed dysphagia after radiotherapy or chemoradiotherapy.
3. Interventions: acupuncture-related interventions used as an adjunct to conventional rehabilitation, supportive care, or other background treatment, including manual acupuncture, electroacupuncture, or other acupoint-based acupuncture techniques.
4. Comparators: control interventions involving the same background treatment as the intervention group but without any form of acupuncture or acupoint stimulation, such as conventional treatment, swallowing rehabilitation, pharmacological treatment, basic supportive care, or other non-acupuncture interventions.
5. Outcomes: at least one of the following outcomes was reported: swallowing-function outcomes, such as the Water Swallowing Test (WST), videofluoroscopic swallowing study (VFSS), or Standardized Swallowing Assessment (SSA); swallowing-related quality-of-life outcomes, such as the MD Anderson Dysphagia Inventory (MDADI) or Swallowing Quality of Life questionnaire (SWAL-QOL); nutritional indicators, such as albumin (ALB) or prealbumin (PA), or safety outcomes, including adverse events.

### Exclusion criteria

1. The participants were not patients with dysphagia after radiotherapy or chemoradiotherapy for nasopharyngeal carcinoma, or relevant data could not be extracted separately.
2. Duplicate publications, in which case only the most complete or most recently published version was retained.
3. The full text was unavailable, or extractable data could not be obtained after contacting the authors.
4. Non-randomized studies, including reviews, systematic reviews, meta-analyses, case reports, conference abstracts, animal studies, and basic research studies.

### Study selection

Retrieved records were imported into NoteExpress for management and duplicate removal. Two reviewers (Z.Y.L. and H.L.S.) independently screened the records. Titles and abstracts were first reviewed to identify potentially eligible studies, and full texts were then assessed to determine final inclusion. Disagreements or uncertainties were resolved through discussion with a third reviewer (Y.H.T.).

### Data extraction

Two reviewers (Z.Y.L. and H.L.S.) independently extracted data from the included studies using a standardized form. The extracted information included the first author, publication year, sample size in each group, participant age and sex, intervention and comparator treatments, and the outcome measures reported. Details of the acupuncture intervention were also recorded, including acupuncture modality, acupoint selection, stimulation method, treatment frequency, treatment duration, and the background treatment provided to both groups. For each outcome, the assessment time point and the numerical data required for meta-analysis, including means and standard deviations or event counts and group totals, were extracted.

When reported, information on treatment completion, adherence to acupuncture and concomitant care, participant withdrawal, reasons for withdrawal, adverse events, and the handling of missing outcome data was also collected. Any disagreement between the two reviewers was resolved through discussion with a third reviewer (Y.H.T.). When relevant information was incomplete or unclear, the corresponding authors were contacted where possible.

### Statistical analysis

Meta-analyses were performed using the meta package in R (version 4.4.0). Dichotomous outcomes were expressed as risk ratios (RRs) with 95% confidence intervals (CIs). For continuous outcomes, mean differences (MDs) were used when outcomes were measured on the same scale, whereas standardized mean differences (SMDs) were used when different measurement scales were applied; corresponding 95% CIs were calculated. Heterogeneity was assessed using Cochran’s Q test and the I² statistic. A fixed-effect model was used when P > 0.05 and I^2^ < 50%; otherwise, a random-effects model was used.

To explore the sources of heterogeneity, exploratory subgroup analyses of post-treatment WST scores were conducted according to treatment duration (≤ 4 weeks vs > 4 weeks) and treatment frequency (< 5 sessions/week vs ≥ 5 sessions/week). These categories were selected according to the distribution of treatment regimens across the included studies. For the clinical effective rate, subgroup analyses were conducted according to whether swallowing rehabilitation was provided as background treatment in both groups and whether local acupoints alone or a combination of local and distal acupoints was used. Differences between subgroups were assessed using a formal test for subgroup differences. Sensitivity analyses were performed using a leave-one-out approach, in which each study was sequentially excluded to assess the robustness of the pooled estimates. The funnel plot, Begg’s rank correlation test and Egger’s regression test were used to assess publication bias, and the trim-and-fill method was employed to correct for potential asymmetry.

### Outcomes

The primary outcomes were swallowing-related measures, including post-treatment Water Swallowing Test (WST) scores, the proportion of patients achieving a favorable swallowing status, clinical effective rate, videofluoroscopic swallowing study (VFSS), and the Standardized Swallowing Assessment (SSA). A post-treatment WST grade of I-II or a negative WST result was considered a favorable swallowing status. The clinical effective rate was based on the combined number of markedly effective and effective cases. Secondary outcomes included swallowing-related quality of life assessed using the MD Anderson Dysphagia Inventory (MDADI) or the Swallowing Quality Of Life Questionnaire (SWAL-QOL), nutritional indicators such as albumin (ALB) and prealbumin (PA), and adverse events.

### Risk of bias

Risk of bias in the included studies was independently assessed by two reviewers (Z.Y.L. and H.L.S.). Disagreements were resolved through discussion with a third reviewer (Y.H.T.). The Cochrane Risk of Bias tool, version 1 (RoB 1), was used to assess the following domains: random sequence generation, allocation concealment, blinding of participants and personnel, blinding of outcome assessment, incomplete outcome data, selective reporting, and other sources of bias. Each domain was judged as having a low, high, or unclear risk of bias, and risk-of-bias graphs were generated.

## Results

### Study selection

A total of 153 records were identified through database searches, and no additional records were identified from other sources. After duplicate records were removed, 89 records remained. Of these, 54 were excluded during title and abstract screening, and 35 full-text articles were assessed for eligibility. After full-text review, 23 articles were excluded for the following reasons: control groups included acupuncture interventions (n = 5), incomplete data descriptions (n = 4), no appropriate control group (n = 8), and full text was unavailable (n = 6). Finally, 12 randomized controlled trials were included in the review (**Figure 1**).

**Figure 1:**
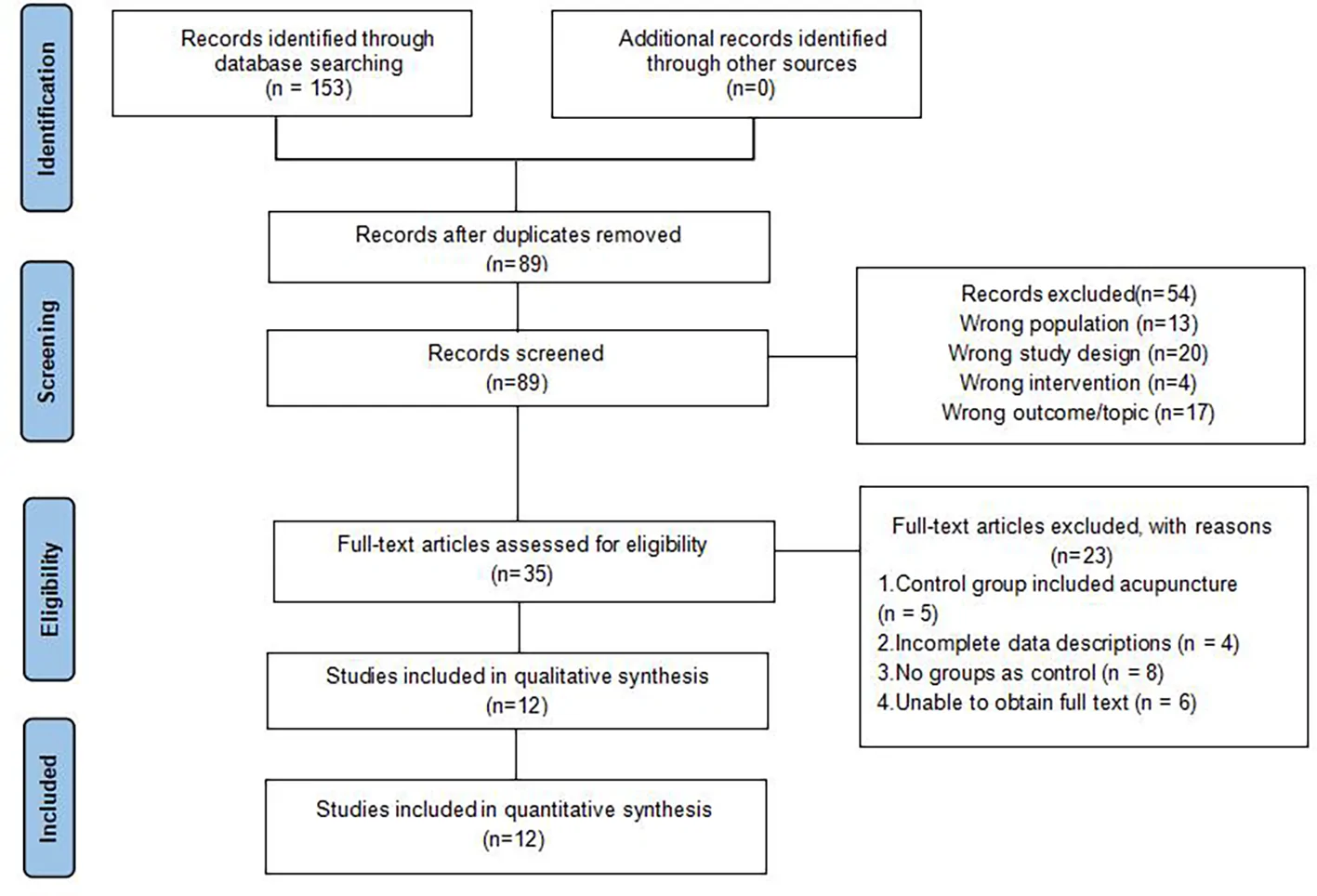
Flowchart of study selection for meta-analysis.

### Characteristics of included studies

A total of 12 randomized controlled trials were included (12–23), all of which were conducted in China. These studies included 863 patients with dysphagia after radiotherapy or chemoradiotherapy for nasopharyngeal carcinoma, with 432 patients in the intervention groups and 431 in the control groups. All but one study reported patient age, and all studies reported sex distribution. Overall, the interventions consisted of acupuncture combined with rehabilitation training, herbal medicine, or basic symptomatic treatment. The control groups received corresponding background treatment without acupuncture or acupoint stimulation. Treatment frequency ranged from once weekly to twice daily, and treatment duration ranged from 1 week to 3 months; one study did not report treatment frequency. Outcomes extracted for this review included post-treatment swallowing-function status, the proportion of patients achieving a favorable swallowing status, clinical effective rate, VFSS, SSA, MDADI, SWAL-QOL, ALB, and PA (**Table 1**).

**Table 1:** Baseline characteristics of the included studies.

First author, year	Group	No. of patients	Age (years)	Sex (M/F)	Treatment	Acupuncture regimen	Outcomes
Gu XJ 2026 (12)	Intervention	30	48.73 ± 4.98	20/10	Acupuncture + ultrasound therapy + conventional rehab. + symptomatic treatment	6 sessions/week, 3 months	WST score; favorable WST status; clinical effective rate; MDADI
	Control	30	48.57 ± 5.06	18/12	Ultrasound therapy + conventional rehab. + symptomatic treatment		
Wu ZP 2025 (13)	Intervention	26	57 (46, 60.25)	18/8	Acupuncture + swallowing rehab. + symptomatic treatment	7 sessions/week, 3 weeks	MDADI
	Control	25	58 (51.5, 60.5)	20/5	Swallowing rehab. + symptomatic treatment		
Hu ZW 2024 (14)	Intervention	30	43.07 ± 7.20	19/11	Acupuncture + CTAR training + conventional rehab.	Once every 2 days, 4 weeks	WST score; favorable WST status; VFSS; SWAL-QOL; ALB; PA
	Control	30	44.13 ± 7.52	22/8	CTAR training + conventional rehab.		
Yang XZ 2023 (15)	Intervention	90	50.79 ± 3.58	62/28	Acupuncture + orofacial rehab. training	2 sessions/week, 2 months	clinical effective rate; SWAL-QOL
	Control	90	51.28 ± 3.46	60/30	Orofacial rehab. training		
Chi FR 2023 (16)	Intervention	30	61.03 ± 2.54	18/12	Electroacupuncture + rehab. training	2 sessions/day, 1 month	favorable WST status
	Control	30	61.25 ± 2.65	20/10	Rehab. training		
Hu ZW 2022 (17)	Intervention	48	56 ± 7	27/21	Acupuncture + sEMG-BFB + Shaker training	6 sessions/week, 2 months	WST score; favorable WST status; VFSS; SWAL-QOL; ALB; PA
	Control	48	56 ± 8	29/19	sEMG-BFB + Shaker training		
Zhao XY 2020 (18)	Intervention	41	43.27 ± 7.19	29/12	Acupuncture + herbal medicine	Every other day, 4 weeks	WST score; clinical effective rate
	Control	42	44.12 ± 7.47	27/15	Herbal medicine		
Huang WC 2018 (19)	Intervention	30	62.00 ± 9.62	23/7	Acupuncture + routine/symptomatic treatment	6 sessions/week, 1 week	WST score; MDADI
	Control	30	58.93 ± 9.52	25/5	Routine/symptomatic treatment		
Deng LY 2016 (20)	Intervention	15	NR	8/7	Acupuncture + herbal medicine + rehab. training	1 session/week, 3 weeks	WST score; VFSS; clinical effective rate
	Control	15	NR	9/6	Herbal medicine + rehab. training		
Hou JY 2015 (21)	Intervention	20	47 ± 6	13/7	Acupuncture + basic treatment/rehab. therapy	5 sessions/week, 4 weeks	WST score; clinical effective rate
	Control	20	48 ± 7	12/8	Basic treatment/rehab. therapy		
Tian SS 2020 (22)	Intervention	41	71.43 ± 3.56	28/13	Acupuncture + rehab. training	NR	SSA; SWAL-QOL
	Control	41	71.42 ± 3.55	27/14	Rehab. training		
Zhang Y 2023 (23)	Intervention	31	56.18 ± 8.57	15/16	Acupuncture + rehab. training	6 sessions/week, 4 weeks	SSA; MDADI; clinical effective rate
	Control	30	56.07 ± 9.12	14/16	Rehab. training		

All included studies were randomized controlled trials. M/F, male/female; T, treatment group; C, control group; WST, Water Swallowing Test; VFSS, videofluoroscopic swallowing study; SSA, Standardized Swallowing Assessment; SWAL-QOL, Swallowing Quality of Life questionnaire; MDADI, M.D. Anderson Dysphagia Inventory; ALB, albumin; PA, prealbumin; CTAR, chin tuck against resistance; sEMG-BFB, surface electromyography biofeedback; NR, not reported; rehab., rehabilitation.

### Risk of bias assessment

The risk-of-bias assessment indicated that the included studies had some methodological limitations, mainly related to incomplete reporting of allocation concealment, blinding, and selective reporting. For random sequence generation, eight studies clearly reported using a random number table, computer-generated randomization, or comparable methods and were judged as having a low risk of bias (13–19, 23). The remaining four studies stated that random allocation was used but did not provide sufficient details and were therefore judged as having an unclear risk of bias (12, 20–22). Allocation concealment was reported in only a few studies (19, 23), whereas most studies did not provide sufficient information and were judged as having an unclear risk of bias (12–18, 20–22). Because of the nature of acupuncture interventions, blinding of participants and personnel was generally difficult to implement, and reporting of blinding of outcome assessment was also limited (12, 14–22). The risk of selective reporting was rated as unclear in all studies, as trial registration information or prespecified protocols were generally unavailable and it was therefore difficult to determine whether all prespecified outcomes had been reported (**Supplementary Figure S1**).

### Swallowing function

#### Post-treatment WST scores

Seven studies reported post-treatment WST scores (12, 14, 17–21), including 429 patients, with 214 in the intervention groups and 215 in the control groups. Heterogeneity testing showed substantial heterogeneity (I² = 64.2%, P = 0.0103). Therefore, a random-effects model was used. The result showed that post-treatment WST scores were lower in the intervention groups than in the control groups (MD = -0.78, 95% CI: -1.04 to -0.51) (**Figure 2A**). In subgroup analyses by treatment duration, the pooled estimates for studies with treatment duration ≤ 4 weeks and > 4 weeks were MD = -0.86 (95% CI: -1.18 to -0.53) and MD = -0.56 (95% CI: -0.84 to -0.27), respectively. Both subgroups favored the intervention groups, but no statistically significant difference was observed between subgroups (P = 0.1761) (**Figure 3A**). In subgroup analyses by treatment frequency, the pooled estimates for < 5 sessions/week and ≥ 5 sessions/week were MD = -0.68 (95% CI: -0.93 to -0.43) and MD = -0.83 (95% CI: -1.26 to -0.41), respectively, with no significant subgroup difference (P = 0.5410) (**Figure 3B**).

**Figure 2:**
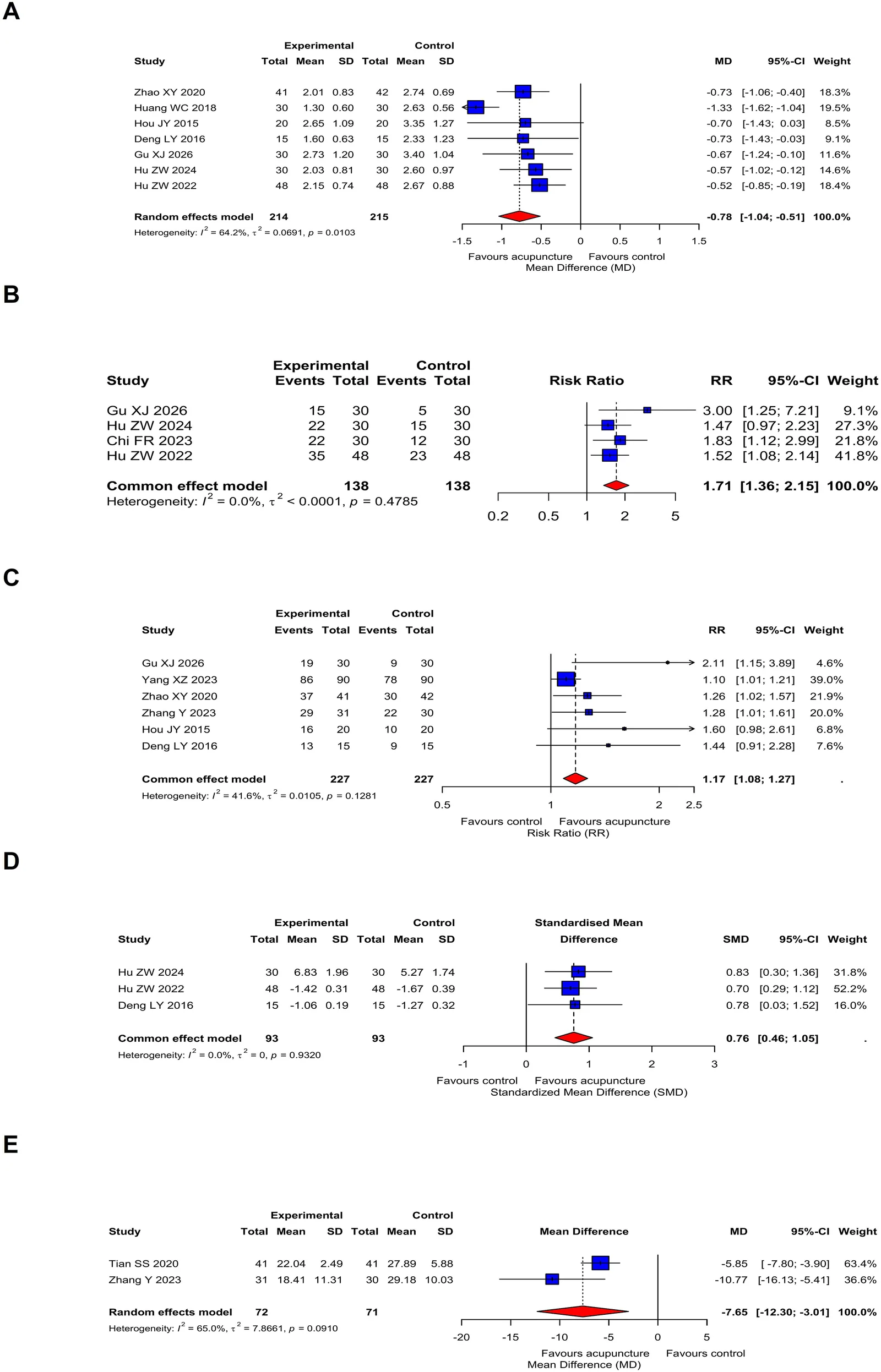
Forest plots of swallowing outcomes: **(A)** post-treatment WST scores, **(B)** proportion of patients achieving favorable swallowing status, **(C)** clinical effective rate, **(D)** VFSS scores, **(E)** SSA scores.

**Figure 3:**
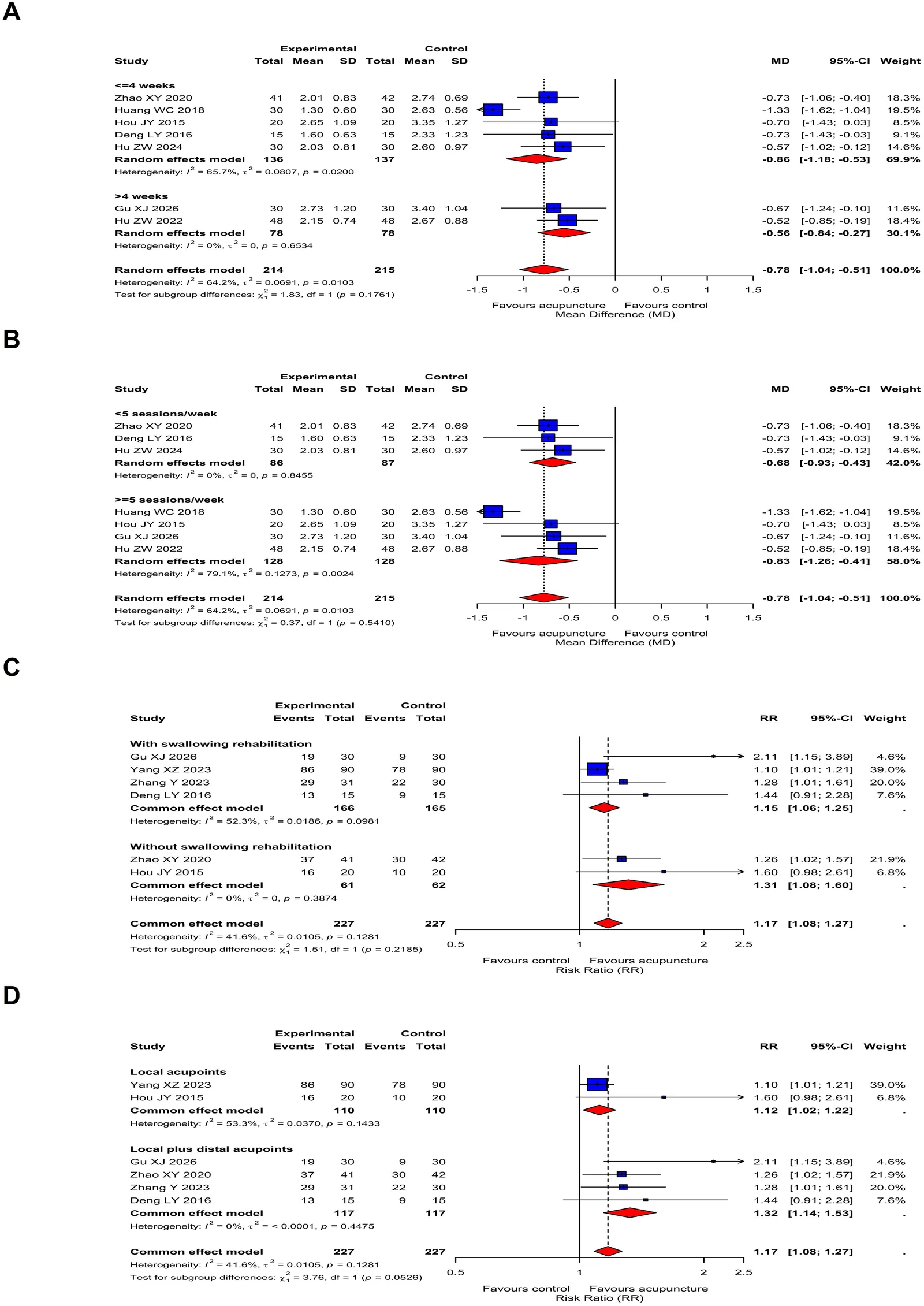
Forest plots of subgroup analyses: **(A)** post-treatment WST scores by treatment duration, **(B)** post-treatment WST scores by treatment frequency, **(C)** clinical effective rate by rehabilitation background, **(D)** clinical effective rate by acupoint selection pattern.

### Proportion achieving favorable swallowing status

Four studies reported the proportion of patients achieving favorable swallowing status after treatment (12, 14, 16, 17), including 276 patients, with 138 in the intervention groups and 138 in the control groups. No obvious heterogeneity was observed (I² = 0%, P = 0.4785). Therefore, a fixed-effect model was used. The result showed that the proportion of patients achieving favorable swallowing status was higher in the intervention groups than in the control groups (RR = 1.71, 95% CI: 1.36 to 2.15) (**Figure 2B**).

### Clinical effective rate

Six studies reported the clinical effective rate (12, 15, 18, 20, 21, 23), including 454 patients, with 227 in the intervention groups and 227 in the control groups. Heterogeneity was low (I² = 41.6%, P = 0.1281). Therefore, a fixed-effect model was used. The result showed that the clinical effective rate was higher in the intervention groups than in the control groups (RR = 1.17, 95% CI: 1.08 to 1.27) (**Figure 2C**). In subgroup analyses by rehabilitation background, the pooled estimates for studies with and without swallowing rehabilitation training were RR = 1.15 (95% CI: 1.06 to 1.25) and RR = 1.31 (95% CI: 1.08 to 1.60), respectively. Both subgroups favored the intervention groups, but the between-subgroup difference was not statistically significant (P = 0.2185) (**Figure 3C**). In subgroup analyses by acupoint selection pattern, the pooled estimates for local acupoint selection and combined local and distal acupoint selection were RR = 1.12 (95% CI: 1.02 to 1.22) and RR = 1.32 (95% CI: 1.14 to 1.53), respectively. Both subgroups favored the intervention groups, and the between-subgroup difference approached but did not reach statistical significance (P = 0.0526) (**Figure 3D**).

### VFSS

Three studies reported VFSS scores (14, 17, 20), including 186 patients, with 93 in the intervention groups and 93 in the control groups. No obvious heterogeneity was observed (I² = 0%, P = 0.9320). Therefore, a fixed-effect model was used. The result showed that VFSS outcomes were better in the intervention groups than in the control groups (SMD = 0.76, 95% CI: 0.46 to 1.05) (**Figure 2D**).

### SSA

Two studies reported SSA scores (22, 23), including 143 patients, with 72 in the intervention groups and 71 in the control groups. Heterogeneity testing indicated substantial heterogeneity (I² = 65.0%, P = 0.0910). Therefore, a random-effects model was used. The result showed that post-treatment SSA scores were lower in the intervention groups than in the control groups (MD = -7.65, 95% CI: -12.30 to -3.01) (**Figure 2E**).

### Quality of life

#### SWAL-QOL

Four studies reported SWAL-QOL scores (14, 15, 17, 22), including 418 patients, with 209 in the intervention groups and 209 in the control groups. Heterogeneity testing indicated substantial heterogeneity (I² = 82.2%, P = 0.0008). Therefore, a random-effects model was used. The result showed that SWAL-QOL scores were higher in the intervention groups than in the control groups (MD = 13.58, 95% CI: 6.50 to 20.65, P = 0.0002) (**Figure 4A**).

**Figure 4:**
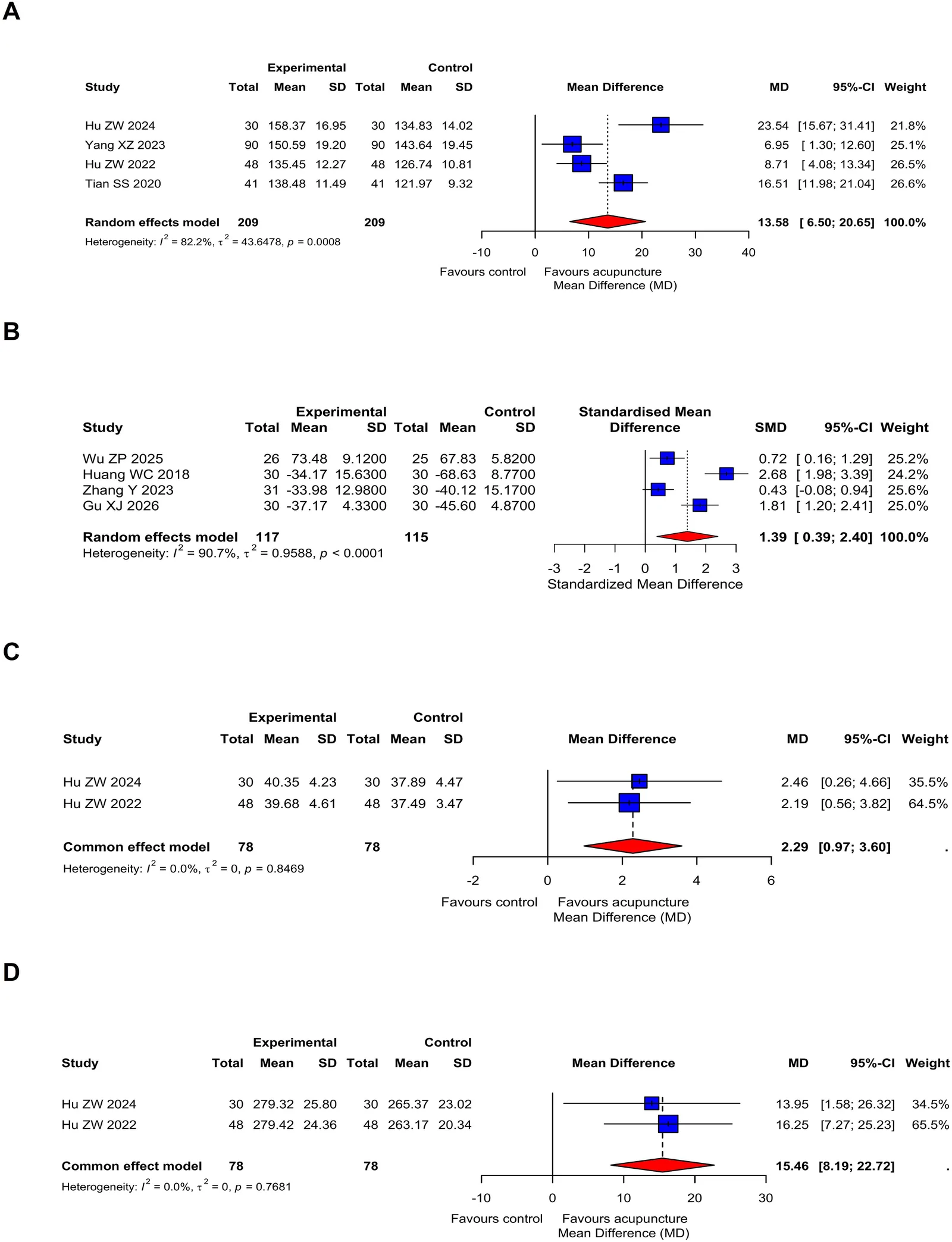
Forest plots of quality-of-life and nutritional outcomes: **(A)** SWAL-QOL scores, **(B)** MDADI scores, **(C)** ALB levels, **(D)** PA levels.

#### MDADI

Four studies reported MDADI scores (12, 13, 19, 23), including 232 patients, with 117 in the intervention groups and 115 in the control groups. Because the direction of MDADI score reporting was not fully consistent across the included studies, effect directions were harmonized before pooling. Heterogeneity testing indicated substantial heterogeneity (I² = 90.7%, P < 0.0001). Therefore, a random-effects model was used. The result showed that MDADI outcomes were better in the intervention groups than in the control groups (SMD = 1.39, 95% CI: 0.39 to 2.40) (**Figure 4B**).

### Nutritional indicators

#### ALB

Two studies reported ALB levels (14, 17), including 156 patients, with 78 in the intervention groups and 78 in the control groups. No obvious heterogeneity was observed (I² = 0%, P = 0.8470). Therefore, a fixed-effect model was used. The result showed that ALB levels were higher in the intervention groups than in the control groups (MD = 2.29, 95% CI: 0.97 to 3.60, P = 0.0006) (**Figure 4C**).

#### PA

Two studies reported PA levels (14, 17), including 156 patients, with 78 in the intervention groups and 78 in the control groups. No obvious heterogeneity was observed (I² = 0%, P = 0.7680). Therefore, a fixed-effect model was used. The result showed that PA levels were higher in the intervention groups than in the control groups (MD = 15.46, 95% CI: 8.19 to 22.72, P < 0.0001) (**Figure 4D**).

### Sensitivity analysis and publication bias

Most outcomes remained stable in the sensitivity analysis. The stability of post-treatment WST scores, favorable swallowing status, the clinical effective rate, and SWAL-QOL was not influenced by the exclusion of any single study, indicating that these outcomes were robust. Although heterogeneity was substantially reduced after omission of individual studies in the WST (19) and clinical effective rate analyses (15), the pooled conclusions remained unchanged. Substantial heterogeneity persisted for SWAL-QOL, but its pooled effect remained statistically significant throughout the leave-one-out analyses. The MDADI result should be interpreted with caution. For MDADI, the pooled effect became non-significant after omitting Gu XJ 2026 (12), with the limited number of studies reporting this outcome contributing to the instability of the result (**Supplementary Figures S2–S6**).

An assessment of publication bias was conducted for the WST scores after treatment. The funnel plot showed a mild asymmetry, but neither the Begg’s test nor the Egger’s test revealed statistically significant asymmetry. The trim-and-fill method suggested the possibility of 3 missing studies, but the corrected pooled effect still supported the acupuncture group, indicating that the potential publication bias did not alter the overall conclusion. Since this outcome included only 7 studies, the above results should be interpreted with caution (**Supplementary Figures S7–S9**).

### Adverse events

A study documented 3 cases of mild acupuncture-related adverse reactions, including 1 case of local hematoma and 2 cases of dizziness and vomiting due to syncope caused by acupuncture. All these conditions improved after simple treatment (18). Another study reported 1 case where a participant withdrew prematurely due to fear of acupuncture, but no obvious adverse reactions were reported (19). The remaining studies did not report safety results.

## Discussion

Previous studies of acupuncture for radiation-related toxicities in head and neck cancer have mainly focused on xerostomia, with some studies suggesting improvements in patient-reported symptoms and salivary function (24). However, current evidence remains insufficient to support the routine use of acupuncture for radiation-related toxicities. Evidence specifically addressing acupuncture for radiation-related dysphagia remains limited.

This meta-analysis investigated the possible adjunctive role of acupuncture in the management of dysphagia after radiotherapy or chemoradiotherapy for nasopharyngeal carcinoma. The findings suggested that acupuncture may be associated with improved post-treatment swallowing status and a higher clinical effective rate. Findings from VFSS and SSA also suggested possible improvements in swallowing dynamics and swallowing safety. In addition, the available evidence provided preliminary indications of potential benefits for quality of life and nutritional indicators.

Dysphagia after radiotherapy or chemoradiotherapy for nasopharyngeal carcinoma reflects impairments across multiple components of swallowing, including oropharyngeal sensation, swallowing responses, bolus propulsion, and airway protection (4, 25). These disturbances may be reflected during water swallowing by repeated swallows or pauses and by coughing or choking (8). The WST findings suggest that acupuncture may help patients complete water swallowing more continuously, with less coughing or choking.

Radiation-induced mucosal injury and salivary hypofunction may reduce bolus lubrication and aggravate swallowing pain and avoidance of oral intake (26, 27). Radiation-associated fibrosis may restrict the movement of the pharyngeal constrictors and tongue base and reduce upper esophageal sphincter opening, thereby impairing bolus propulsion and pharyngeal clearance (3–5). The favorable VFSS findings may therefore reflect more efficient pharyngeal bolus transit and better overall swallowing performance.

Beyond bolus transit, airway protection is another important component of swallowing function (28). Glossopharyngeal and vagal involvement was commonly accompanied by dysphagia and coughing, whereas hypoglossal involvement was characterized primarily by tongue weakness (29). Another study reported that vagal or recurrent laryngeal nerve palsy was associated with abnormal glottic closure, choking, and aspiration (30). The SSA may therefore reflect better overall clinical swallowing performance, including fewer signs of impaired swallowing safety.

Acupuncture may also have benefits for quality of life. Dysphagia can increase the burden of eating and further affect oral intake, eating in social settings, and daily activities. The SWAL-QOL findings suggested that possible improvements in swallowing status may be accompanied by reductions in eating discomfort, avoidance of oral intake, and limitations in social functioning. The MDADI findings suggested a possible improvement in the quality of life after acupuncture treatment; however, this outcome was based on a small number of studies, showed substantial heterogeneity, and demonstrated limited stability in the sensitivity analysis. These limitations highlight the necessity of more standardized and consistent collection of quality-of-life data in future trials. Future research should use appropriate swallowing quality of life scales for assessment, evaluate the results at pre-defined and comparable time points, fully report the data and transparently handle missing data, and incorporate long-term follow-up assessments.

Although the findings suggest a potential benefit of acupuncture for dysphagia, the underlying mechanisms remain uncertain. Several potential mechanisms may contribute to these effects: Firstly, acupuncture may act on surrounding tissues through local neurovascular and neuromuscular responses. A study found that electroacupuncture stimulation at the CV23 acupoint could increase the expression of TRPV1, calcitonin gene-related peptide, and tyrosine hydroxylase near the acupoint, and promote local tissue perfusion (31). Cui et al. further reported that electroacupuncture could increase local muscle blood flow, increase hypoglossal motor conduction velocity, and enhance the activity of the tongue muscles (32). These changes may be beneficial for nerve conduction and muscle contraction. Second, acupuncture may promote central sensorimotor integration through multiple interconnected swallowing-related neural pathways. Studies have shown that electroacupuncture at CV23 can engage a pathway connecting the nucleus tractus solitarius (NTS), ventral posteromedial thalamic nucleus (VPM), and primary somatosensory cortex (S1), thereby facilitating the transmission of peripheral sensory input to cortical sensory regions (33). CV23 stimulation also activates excitatory neurons in the paraventricular hypothalamus (PVH), which project to the parabrachial nucleus (PBN) and NTS and may provide upstream modulatory input to the brainstem swallowing network (11). Electroacupuncture at CV23 can modulate swallowing interneurons in the ventrolateral medulla and increase swallowing activity (34). In a separate study, combined stimulation of GV16 and CV23 increased 5-HT1A expression in the NTS, while blockade of 5-HT1A attenuated the swallowing response (35). These findings suggest that acupuncture may enhance the transmission and integration of swallowing sensory input and facilitate coordinated swallowing control (36, 37). Third, acupuncture may improve the peripheral biomechanics of swallowing impaired after radiotherapy. Radiation-induced fibrosis and neuromuscular injury can impair pharyngeal contraction, hyolaryngeal excursion, and bolus clearance, thereby contributing to persistent swallowing dysfunction (3, 5, 8). Tongue needle acupuncture produced favorable changes in oral transit, soft-palate elevation, hyoid displacement, and upper esophageal phincter opening compared with routine rehabilitation (23). One study found that acupuncture combined with swallowing rehabilitation produced a greater reduction in genioglossus Young’s modulus, together with greater improvements in penetration-aspiration, oral transit time, and pharyngeal constriction (13). These findings suggest that acupuncture may influence both the mobility and stiffness of swallowing tissues, although whether it can reverse established radiation-induced fibrosis remains uncertain. Fourth, acupuncture may help mitigate oral dysfunction associated with radiation-induced salivary gland injury. Radiotherapy damages salivary acinar cells and impairs water transport, resulting in reduced salivary secretion and persistent xerostomia (38, 39). Electroacupuncture treatment reduced the expression of the pro-inflammatory cytokines TNF-α and IL-1β in mice with chemotherapy-induced salivary gland hypofunction, increased AQP5 expression, preserved salivary acinar structure, and increased salivary secretion (40). Acupuncture may also promote residual salivary gland secretion through neuropeptide-mediated neurovascular regulation. Acupuncture has been shown to increase vasoactive intestinal polypeptide (VIP) and calcitonin gene-related peptide (CGRP) levels in patients with xerostomia, which may enhance glandular blood flow and facilitate the secretory response of remaining functional salivary tissue (41, 42). Increased salivary secretion may improve oral lubrication and bolus formation, thereby facilitating oral transport during swallowing (**Figure 5****).**

**Figure 5:**
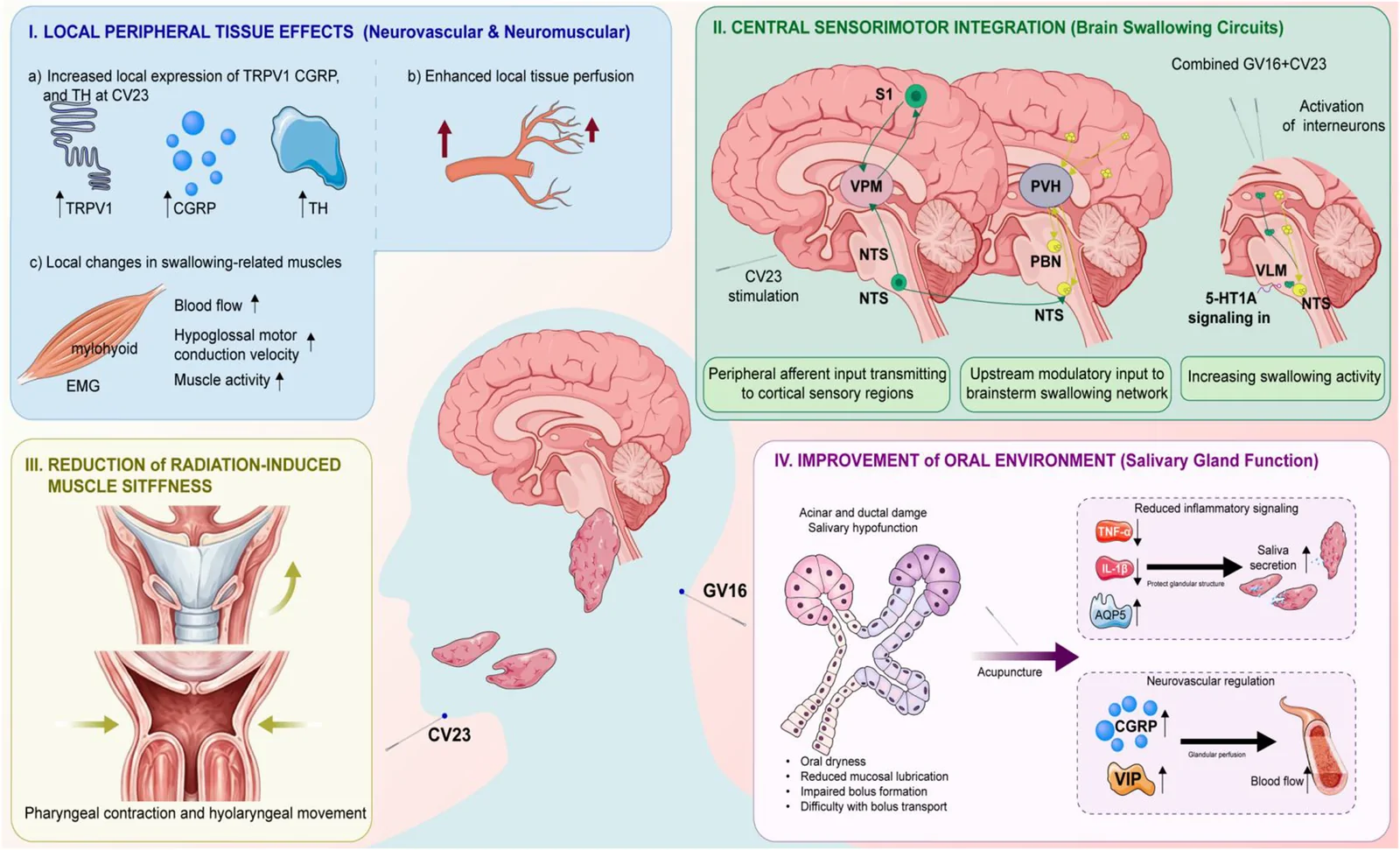
The mechanism diagram of dysphagia after radiotherapy or chemoradiotherapy in nasopharyngeal carcinoma and the mechanism of acupuncture in improving dysphagia.

Safety and tolerability are important considerations in the clinical use of acupuncture. Evidence from oncology trials suggests that acupuncture is not associated with an increased risk of serious adverse events compared with sham acupuncture or active controls, although nonserious events such as local pain, minor bleeding, bruising or hematoma, and vegetative reactions may occur (43). In the randomized controlled trials included in this review, safety data were sparsely reported. Among the few trials reporting safety outcomes, the documented acupuncture adverse events were mild, mainly including local hematoma and discomfort related to fainting during acupuncture, and no serious consequences were reported after simple management. However, the sparse and incomplete reporting of adverse events precludes firm conclusions regarding the safety and tolerability of acupuncture in this population. Future trials should systematically monitor and report adverse events.

Substantial heterogeneity was an important consideration in this review. Subgroup analyses examining treatment duration, treatment frequency, rehabilitation background, and acupoint selection did not identify a clear source of heterogeneity. In the WST analysis, the exclusion of one study substantially reduced heterogeneity without changing the direction of the effect (19). Variation in the interval since radiotherapy or chemoradiotherapy may have contributed to differences in treatment response across studies. In the earlier post-treatment phase, improvement may partly coincide with the resolution of acute mucosal reactions, pain, or xerostomia, whereas later fibrotic and neuropathic changes may be less reversible. Baseline swallowing function may also have influenced the observed effect. Patients with milder impairment may have had less room for measurable improvement, whereas advanced fibrosis or cranial nerve injury may have limited the potential for recovery. Other differences in acupuncture delivery, such as acupuncture modality and stimulation method, may also have contributed to the variation across studies.

Although randomization is intended to balance prognostic factors between groups, most included trials were small, and allocation concealment was incompletely reported. It therefore remained uncertain whether important factors such as baseline dysphagia severity, time since radiotherapy or chemoradiotherapy, concurrent chemotherapy, and nutritional status were adequately balanced between groups. Potential imbalances in these factors could have influenced swallowing recovery and the observed effect of acupuncture.

Adherence and attrition were not consistently reported across the included studies. Although most studies described the planned frequency and duration of acupuncture, few reported the actual number of sessions completed or participants’ adherence to rehabilitation and care. Moreover, differences in adherence to acupuncture and background rehabilitation may have influenced the observed treatment effect. Because reasons for withdrawal and the handling of missing outcome data were not consistently reported, the potential influence of attrition on the effect estimates could not be fully assessed. Future trials should report both planned and completed treatment sessions, adherence to concomitant rehabilitation, reasons for withdrawal, and the methods used to handle treatment interruptions and missing data.

Acupuncture has a long history of clinical use in China and has developed a relatively mature education, training and practice system. In contrast, in countries where acupuncture has not yet been widely integrated into clinical practice, patients and healthcare professionals may have different levels of familiarity, awareness and acceptance of acupuncture. The training background and clinical experience of acupuncture practitioners may also vary. Studies have shown that there are significant differences in service models, professional training, cultural acceptance and healthcare system support for complementary therapies such as acupuncture among different countries (44). Therefore, the acupuncture protocols and clinical effects adopted in clinical research conducted in China may not be implemented and reproduced in other healthcare settings with the same certainty. Another survey found that medical staff lack knowledge, experience and confidence in using complementary therapies such as acupuncture, as well as a clear referral pathway, which may affect the implementation of related therapies in routine cancer supportive care; insufficient insurance coverage and high out-of-pocket costs were also identified as potential barriers (45). Although these differences may limit the generalizability of current research results, dysphagia after radiotherapy or chemoradiotherapy also constitutes an important clinical burden in other countries, and the existing rehabilitation and supportive care still cannot enable all patients to fully recover. Therefore, in the future, rigorous multicenter randomized controlled trials with well-designed protocols need to be conducted in different countries and medical systems to determine whether acupuncture can achieve similar clinical benefits and further evaluate its acceptability and clinical feasibility.

This review has several limitations. First, although all included studies were randomized controlled trials, most had small sample sizes, and blinding of practitioners and participants was often difficult because of the nature of acupuncture interventions. The reporting of important prognostic factors was limited, including baseline dysphagia severity, time since radiotherapy or chemoradiotherapy, radiation exposure and nutritional status. Imbalances in these factors may have influenced swallowing recovery independently of acupuncture. Second, several secondary outcomes were informed by only a few small studies, limiting the precision of the estimates and precluding reliable assessment of publication bias. Adverse-event reporting was also incomplete, limiting conclusions regarding safety. Some outcomes may therefore have been affected by small-study effects or potential publication bias. Third, most studies assessed only short-term changes at the end of treatment and lacked long-term follow-up of swallowing function, aspiration risk, nutritional status, and quality of life.

## Conclusions

The current evidence suggests that acupuncture may have a potential adjunctive role in the management of dysphagia after radiotherapy or chemoradiotherapy for nasopharyngeal carcinoma, with possible benefits for swallowing function, quality of life, and nutritional status. Larger, rigorously designed randomized controlled trials are needed to further evaluate its efficacy and safety in this setting.

## Data Availability

The raw data supporting the conclusions of this article will be made available by the authors, without undue reservation.
