# Supplementary material for: Efficacy and safety of acupuncture for dysphagia after radiotherapy or chemoradiotherapy in patients with nasopharyngeal carcinoma: a systematic review and meta-analysis
Source: Front Oncol. 2026 Sep 24;16:1919320. doi: 10.3389/fonc.2026.1919320 (PMC13645590; doi:10.3389/fonc.2026.1919320)
Supplement: Supplementary file 1 [file DataSheet1.pdf]

Supplementary Table S1: The search strategy for the respective database.

Database name	Search strategies
CNKI	TKA%=('头颈部肿瘤'+'头颈部癌'+'鼻咽癌'+'鼻咽部癌'+'鼻咽肿瘤'+'鼻咽恶性肿瘤'+'鼻咽部肿瘤'+'耳鼻咽喉肿瘤') AND TKA%=('放疗'+'放射治疗'+'放疗后'+'放射治疗后'+'放化疗'+'放化疗后'+'放射化疗'+'同步放化疗') AND TKA%=('吞咽障碍'+'吞咽困难'+'吞咽功能障碍'+'吞咽功能受损'+'咽下障碍'+'口咽性咽下困难'+'食管咽下困难') AND TKA%=('针灸'+'针刺'+'毫针'+'电针'+'舌三针'+'舌针'+'项针'+'颊针'+'浮针'+'温针灸')
Wanfang	主题:("鼻咽癌" or "鼻咽部癌" or "鼻咽肿瘤" or "鼻咽恶性肿瘤" or "鼻咽部恶性肿瘤" or "鼻咽部肿瘤" or "头颈部肿瘤" or "头颈部癌" or "耳鼻咽喉肿瘤")<br>And 主题:("放疗" or "放射治疗" or "放疗后" or "放射治疗后" or "放化疗" or "放化疗后" or "放射化疗" or "同步放化疗") and 主题:("吞咽障碍" or "吞咽困难" or "吞咽功能障碍" or "吞咽功能受损" or "吞咽功能" or "咽下障碍" or "口咽性咽下困难" or "食管咽下困难") and 主题:("针灸" or "针刺" or "毫针" or "电针" or "舌三针" or "舌针" or "项针" or "颊针" or "浮针" or "温针灸")
VIP	主题:("鼻咽癌" or "鼻咽部癌" or "鼻咽肿瘤" or "鼻咽恶性肿瘤" or "鼻咽部恶性肿瘤" or "鼻咽部肿瘤" or "头颈部肿瘤" or "头颈部癌" or "耳鼻咽喉肿瘤")<br>and 主题:("放疗" or "放射治疗" or "放疗后" or "放射治疗后" or "放化疗" or "放化疗后" or "放射化疗" or "同步放化疗") and 主题:("吞咽障碍" or "吞咽困难" or "吞咽功能障碍" or "吞咽功能受损" or "吞咽功能" or "咽下障碍" or "口咽性咽下困难" or "食管咽下困难") and 主题:("针灸" or "针刺" or "毫针" or "电针" or "舌三针" or "舌针" or "项针" or "颊针" or "浮针" or "温针灸")
SinoMed	#1：鼻咽癌[不加权:扩展]<br>#2: 头颈部肿瘤[常用字段:智能] OR 头颈部癌[常用字段:智能] OR 鼻咽癌[常用字段:智能] OR 鼻咽部癌[常用字段:智能] OR 鼻咽肿瘤[常用字段:智能] OR 鼻咽恶性肿瘤[常用字段:智能] OR 鼻咽部肿瘤[常用字段:智能] OR 耳鼻咽喉肿瘤[常用字段:智能]<br>#3：#1 OR #2<br>#4："吞咽障碍"[不加权:扩展]<br>#5: 吞咽障碍[常用字段:智能] OR 吞咽困难[常用字段:智能] OR 吞咽功能障碍[常用字段:智能] OR 吞咽功能受损[常用字段:智能] OR 咽下障碍[常用字段:智能] OR 口咽性咽下困难[常用字段:智能] OR 食管咽下困难[常用字段:智能]<br>#6：#4 OR #5<br>#7："针灸"[不加权:扩展]<br>#8：针灸[常用字段:智能] OR 针刺[常用字段:智能] OR 毫针[常用字段:智能] OR 电针[常用字段:智能] OR 舌三针[常用字段:智能] OR 舌针[常用字段:智能] OR 项针[常用字段:智能] OR 颊针[常用字段:智能] OR 浮针[常用字段:智能] OR 温针灸[常用字段:智能]<br>#9：#7 OR #8

	#10：#3 AND #6 AND #9
PubMed	#1: ("Nasopharyngeal Neoplasms"[MeSH Terms]) OR (Nasopharyngeal Carcinoma) OR (Nasopharyngeal Carcinomas) OR (Nasopharyngeal Cancer) OR (Nasopharyngeal Cancers) OR (Nasopharyngeal Neoplasm) OR (Nasopharyngeal Neoplasms) OR (Nasopharyngeal Tumor) OR (Nasopharyngeal Tumors) OR (NPC)<br>#2：("Deglutition Disorders"[MeSH Terms]) OR (Dysphagia) OR ("Swallowing Disorder") OR ("Swallowing Disorders") OR ("Swallowing Dysfunction") OR ("Swallowing Dysfunctions") OR ("Deglutition Disorder") OR ("Deglutition Disorders") OR ("Swallowing Difficulty") OR ("Swallowing Difficulties") OR ("Impaired Swallowing") OR ("Swallowing Impairment") OR ("Swallowing Function")<br>#3：("Acupuncture Therapy"[MeSH Terms]) OR ("Acupuncture"[MeSH Terms]) OR (Acupuncture) OR ("Acupuncture Therapy") OR ("Acupuncture Treatment") OR (Needling) OR ("Manual Acupuncture") OR (Electroacupuncture) OR ("Electro-acupuncture") OR ("Auricular Acupuncture") OR ("Tongue Acupuncture") OR ("Neck Acupuncture")<br>#4：("Radiotherapy"[MeSH Terms]) OR ("Drug Therapy"[MeSH Terms]) OR (Radiotherapy) OR ("Radiation Therapy") OR (Irradiation) OR (Chemotherapy) OR (Chemoradiotherapy) OR ("Chemo-radiotherapy") OR ("Concurrent Chemoradiotherapy") OR (CCRT)<br>#5：#1 AND #2 AND #3 AND #4
Cochrane Library	#1 MeSH descriptor: [Nasopharyngeal Neoplasms] explode all trees<br>#2 Nasopharynx Cancer OR Nasopharyngeal Cancer OR Cancer of Nasopharynx OR Cancers, Nasopharyngeal OR Cancers, Nasopharynx OR Cancer, Nasopharynx OR Cancer, Nasopharyngeal OR Nasopharynx Cancers OR Nasopharyngeal Cancers OR Cancer of the Nasopharynx OR Nasopharynx Neoplasm OR Neoplasms, Nasopharynx OR Nasopharyngeal Neoplasm OR Neoplasm, Nasopharyngeal OR Neoplasms, Nasopharyngeal OR Neoplasm, Nasopharynx OR Nasopharynx Neoplasms<br>#3 #1 OR #2<br>#4 MeSH descriptor: [Deglutition Disorders] explode all trees<br>#5 Dysphagia OR Disorders, Deglutition OR Swallowing Disorders OR Swallowing Disorder OR Deglutition Disorder OR Dysphagia, Esophageal OR Esophageal Dysphagia OR Oropharyngeal Dysphagia OR Dysphagia, Oropharyngeal<br>#6 #4 OR #5<br>#7 MeSH descriptor: [Acupuncture] explode all trees<br>#8 Pharmacopuncture OR Acupuncture Therapy OR Acupuncture OR Acupuncture OR Acupuncture Therapy OR Acupuncture Treatment OR Needling OR Manual Acupuncture OR Electroacupuncture OR Electro-acupuncture OR Auricular Acupuncture OR Tongue Acupuncture OR Neck Acupuncture<br>#9 #7 OR #8<br>#10 MeSH descriptor: [Radiotherapy] explode all trees<br>#11 Radiation Therapy, Targeted OR Targeted Radiation Therapy OR Targeted Radiotherapy OR Targeted Radiation Therapies OR Radiotherapies, Targeted OR Therapies, Targeted Radiation OR Radiation Therapies, Targeted OR Radiotherapy, Targeted OR Therapy, Targeted Radiation OR Targeted Radiotherapies OR Therapies, Radiation OR Radiotherapies OR Radiation Treatments OR Radiation Therapy OR Therapy, Radiation OR Radiation Treatment OR Treatment, Radiation OR Radiation Therapies

	#12 #10 OR #11<br>#13 #3 AND #6 AND #9 AND #12
Web of Science	#1：TS= (Nasopharyngeal Neoplasms OR Nasopharyngeal Carcinoma OR Nasopharyngeal Carcinomas OR Nasopharyngeal Cancer OR Nasopharyngeal Cancers OR Nasopharyngeal Neoplasm OR Nasopharyngeal Neoplasms OR Nasopharyngeal Tumor* OR NPC)<br>#2：TS= (Deglutition disorders OR Dysphagia OR Swallowing Disorder OR Swallowing Disorders OR Swallowing Dysfunction OR Swallowing Dysfunctions OR Deglutition Disorder OR Deglutition Disorders OR Swallowing Difficulty OR Swallowing Difficulties OR Impaired Swallowing OR Swallowing Impairment OR Swallowing Function)<br>#3：TS= (Acupuncture Therapy OR Acupuncture OR Acupuncture OR Acupuncture Therapy OR Acupuncture Treatment OR Needling OR Manual Acupuncture OR Electroacupuncture OR Electro-acupuncture OR Auricular Acupuncture OR Tongue Acupuncture OR Neck Acupuncture)<br>#4：TS= (Radiotherapy OR Drug Therapy OR Radiotherapy OR Radiation Therapy OR Irradiation OR Chemotherapy OR Chemoradiotherapy OR Chemo-radiotherapy OR Concurrent Chemoradiotherapy OR CCRT)<br>#5：#1 AND #2 AND #3 AND #4
Embase	#1 'nasopharynx tumor'/exp<br>#2 'epipharynx tumor':ab,ti OR 'epipharynx tumour':ab,ti OR 'nasopharyngeal neoplasm':ab,ti OR 'nasopharyngeal neoplasms':ab,ti OR 'nasopharyngeal tumor':ab,ti OR 'nasopharyngeal tumour':ab,ti OR 'nasopharynx neoplasm':ab,ti OR 'nasopharynx tumour':ab,ti OR 'neoplasm of the nasopharynx':ab,ti OR 'rhinopharyngeal tumor':ab,ti OR 'rhinopharynx tumor':ab,ti OR 'rhinopharynx tumour':ab,ti OR 'tumor of the epipharynx':ab,ti OR 'tumor of the nasopharynx':ab,ti OR 'tumor of the rhinopharynx':ab,ti OR 'nasopharynx tumor':ab,ti<br>#3 #1 OR #2<br>#4 'dysphagia'/exp<br>#5 aphagopraxia:ab,ti OR 'deglutition difficulty':ab,ti OR 'deglutition disorder':ab,ti OR 'deglutition disorders':ab,ti OR 'difficult deglutition':ab,ti OR 'difficulty in swallowing':ab,ti OR 'difficulty swallowing':ab,ti OR dysphagia:ab,ti OR dysphagias:ab,ti OR 'swallowing difficult':ab,ti OR 'swallowing difficultness':ab,ti OR 'swallowing difficulty':ab,ti OR 'swallowing disorder':ab,ti OR dysphagia:ab,ti<br>#6 #4 OR #5<br>#7 'acupuncture'/exp<br>#8 'acupuncture therapy'/exp OR 'acupuncture'/exp OR acupuncture:ab,ti OR 'acupuncture therapy':ab,ti OR 'acupuncture treatment':ab,ti OR needling:ab,ti OR 'manual acupuncture':ab,ti OR electroacupuncture:ab,ti OR 'electro-acupuncture':ab,ti OR 'auricular acupuncture':ab,ti OR 'tongue acupuncture':ab,ti OR 'neck acupuncture':ab,ti<br>#9 #7 OR #8<br>#10 'chemotherapy'/exp<br>#11 'chemotherapeutic medication':ab,ti OR 'chemotherapeutic

treatment':ab,ti OR chemotherapeutics:ab,ti OR chemotherapy:ab,ti
#12 #10 OR #11
#13 #3 AND #6 AND #9 AND #12

CNKI: China National Knowledge Infrastructure; SinoMed: the Chinese Biomedical Literature Database; WanFang: the WanFang Database; VIP: the Chinese Scientific Journals Full-Text Database;Embase Database; Excerpta Medica Database; WOS Database: Web of Science Database;PubMed Database: the PubMed Database.

Post-treatment WST scores

Omitting	MD	95%-CI	Heterogeneity (I^2)
Zhao XY 2020	-0.78	-1.09 to -0.47	69.4%
**Huang WC 2018**	**-0.63**	**-0.81 to -0.45**	**0%**
Hou JY 2015	-0.78	-1.07 to -0.50	69.9%
Deng LY 2016	-0.78	-1.06 to -0.49	70.0%
Hu ZW 2024	-0.81	-1.10 to -0.52	67.5%
Gu XJ 2026	-0.79	-1.08 to -0.49	69.6%
Hu ZW 2022	-0.83	-1.12 to -0.54	59.8%
Pooled estimate	-0.78	-1.04 to -0.51	64.2%

Favorable swallowing status

Omitting	RR	95%-CI	Heterogeneity (I^2)
Gu XJ 2026	1.58	1.25 to 1.99	0%
Hu ZW 2024	1.80	1.37 to 2.36	5.0%
Chi FR 2023	1.67	1.29 to 2.17	10%
Hu ZW 2022	1.84	1.36 to 2.51	7.8%
Pooled estimate	1.71	1.36 to 2.15	0%

Clinical Effective Rate

Omitting	RR	95%-CI	Heterogeneity (I^2)
Gu XJ 2026	1.16	1.07 to 1.25	18.7%
**Yang XZ 2023**	**1.34**	**1.17 to 1.55**	**0%**
Zhao XY 2020	1.16	1.07 to 1.26	50.1%
Zhang Y 2023	1.16	1.07 to 1.26	49.9%
Hou JY 2015	1.16	1.07 to 1.26	42.5%
Deng LY 2016	1.16	1.07 to 1.26	48.2%
Pooled estimate	1.17	1.08 to 1.27	41.6%

SWAL-QOL

Omitting	MD	95%-CI	Heterogeneity (I^2)
Hu ZW 2024	10.87	5.06 to 16.67	76.7%
Yang XZ 2023	15.79	7.67 to 23.92	83.1%
Hu ZW 2022	15.39	6.24 to 24.54	84.2%
Tian SS 2020	12.67	2.80 to 22.55	84.2%
Pooled estimate	13.58	6.50 to 20.65	82.2%

MDADI

Omitting	SMD	95%-CI	Heterogeneity (I^2)
Wu ZP 2025	1.62	0.33 to 2.91	92.9%
Huang WC 2018	0.97	0.16 to 1.79	83.6%
Zhang Y 2023	1.72	0.61 to 2.83	89.2%
Gu XJ 2026	1.26	**-0.11 to 2.63**	92.7%
Pooled estimate	1.39	0.39 to 2.40	90.7%

Supplementary Table S2: Sensitivity analysis table for Post-treatment WST scores, favorable swallowing status, Clinical Effective Rate, SWAL-QOL, MDADI. The bold lines indicate that after the corresponding studies were conducted, the heterogeneity among the studies or the statistical significance of the combined effect changed.

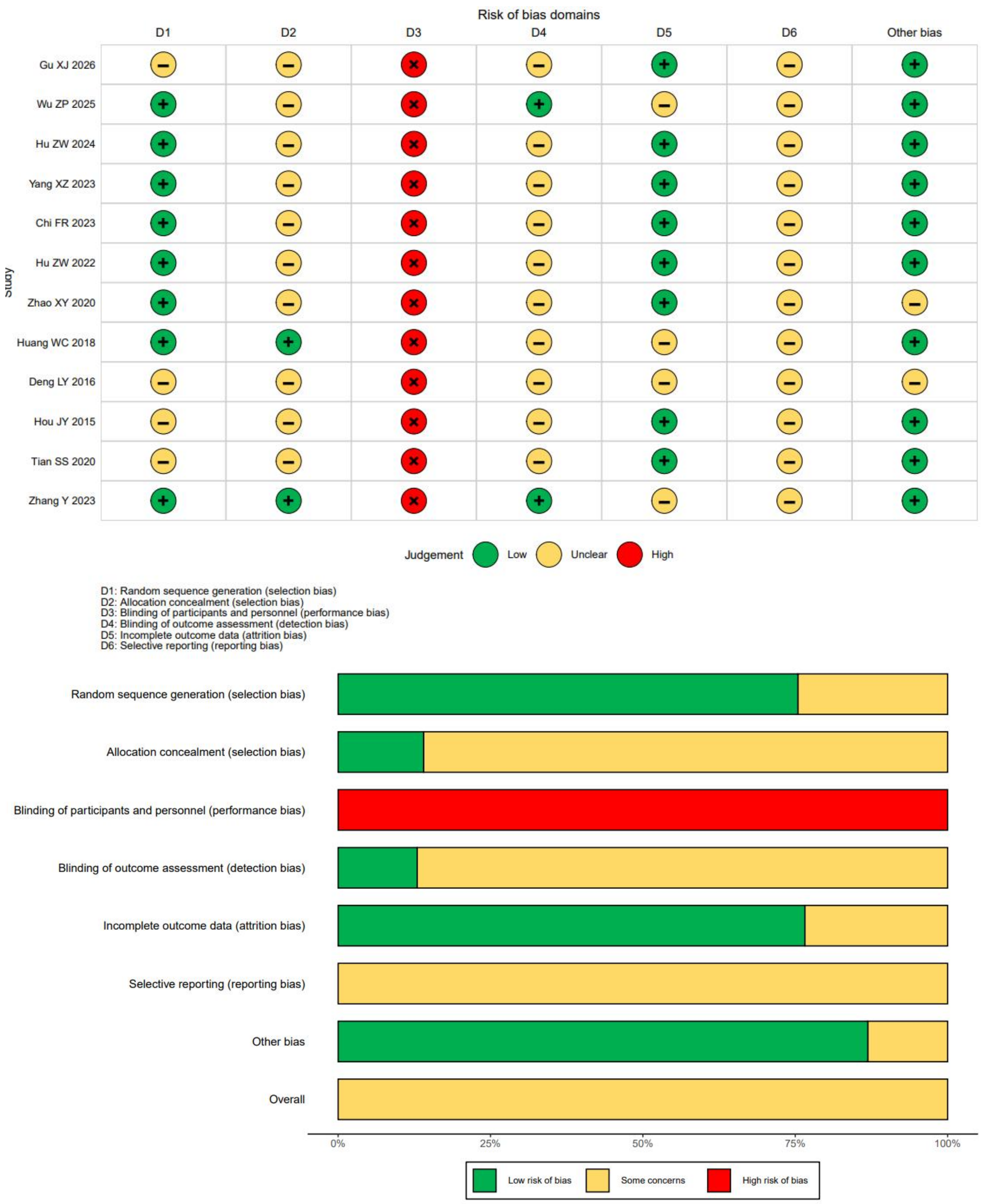

Supplementary Figure S1: Risk of bias of the included randomized controlled trials assessed using the Cochrane ROB 1 tool.

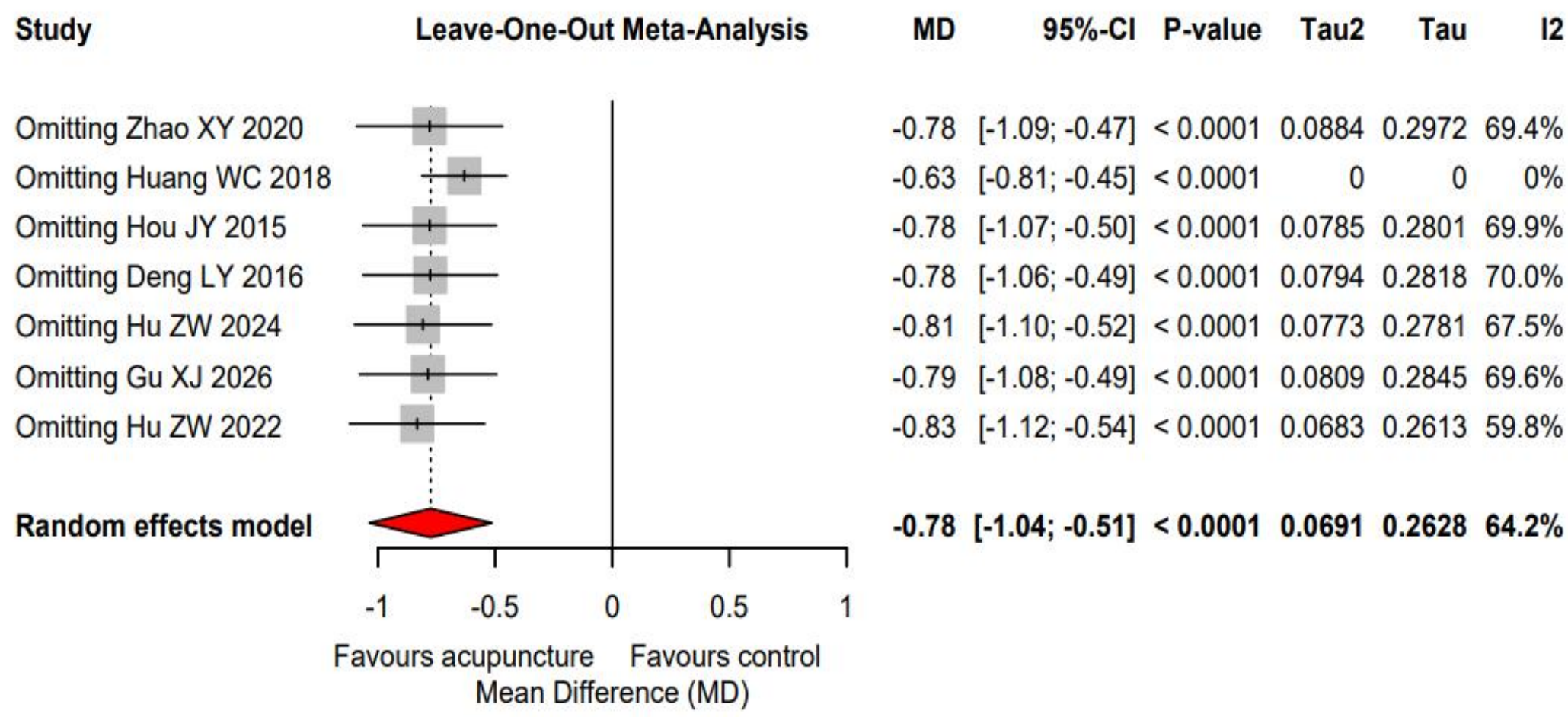

Supplementary Figure S2: Leave-one-out sensitivity analysis for Post-treatment WST scores.

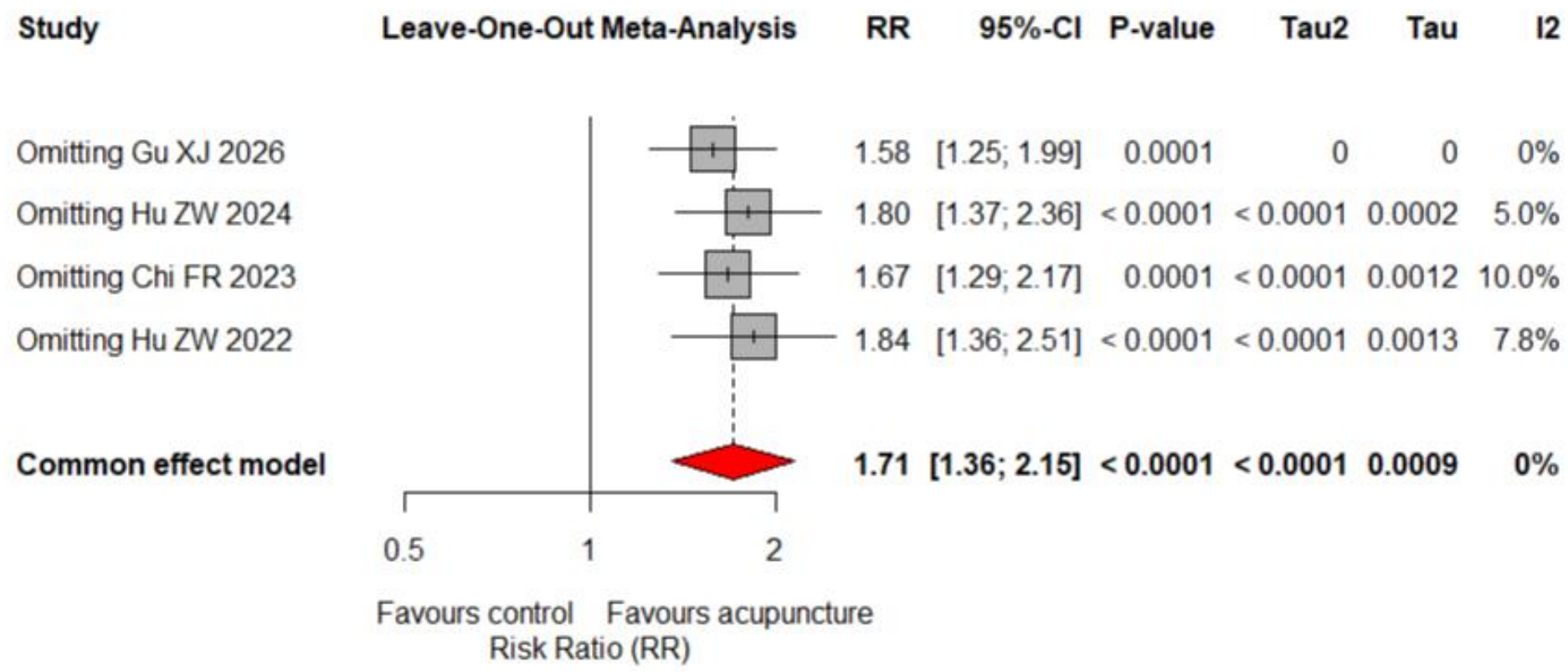

Supplementary Figure S3: Leave-one-out sensitivity analysis for Favorable swallowing status.

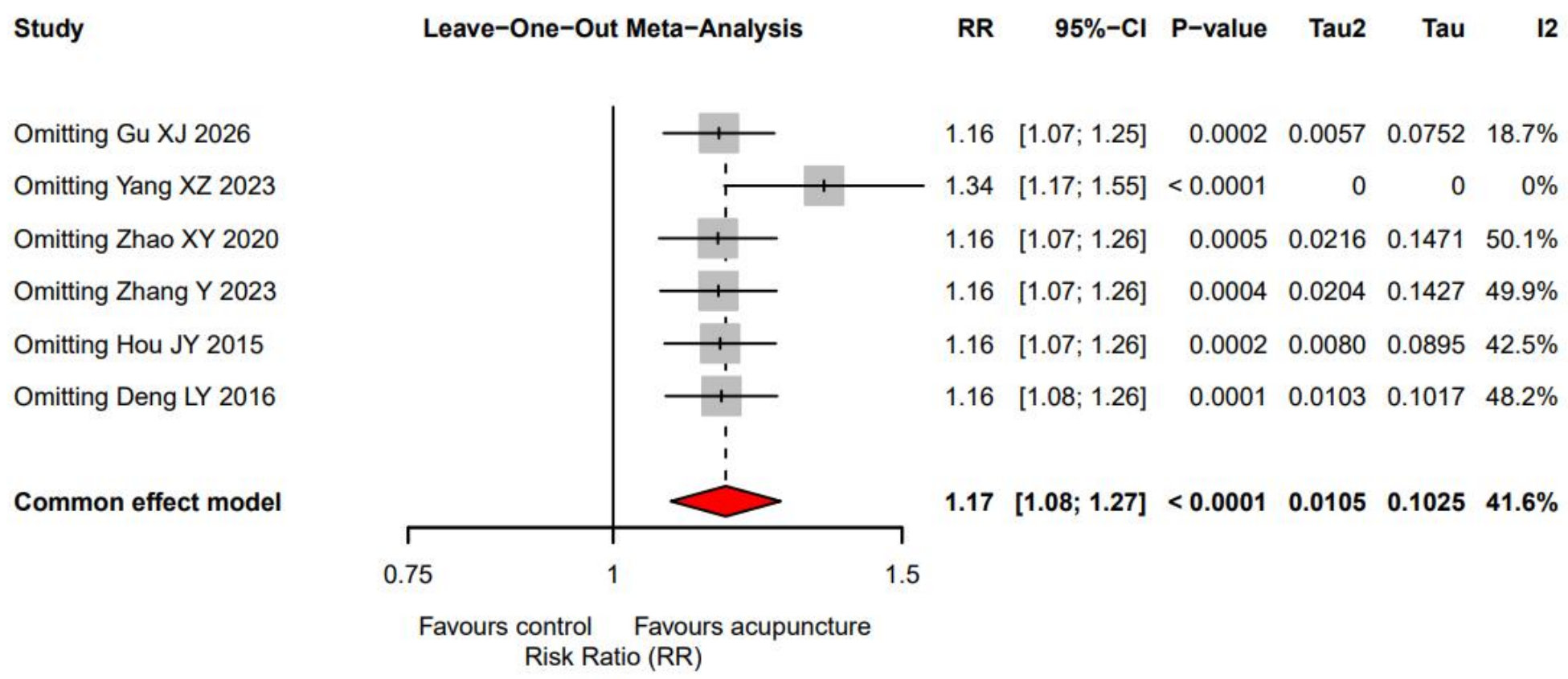

Supplementary Figure S4: Leave-one-out sensitivity analysis for Clinical Effective Rate.

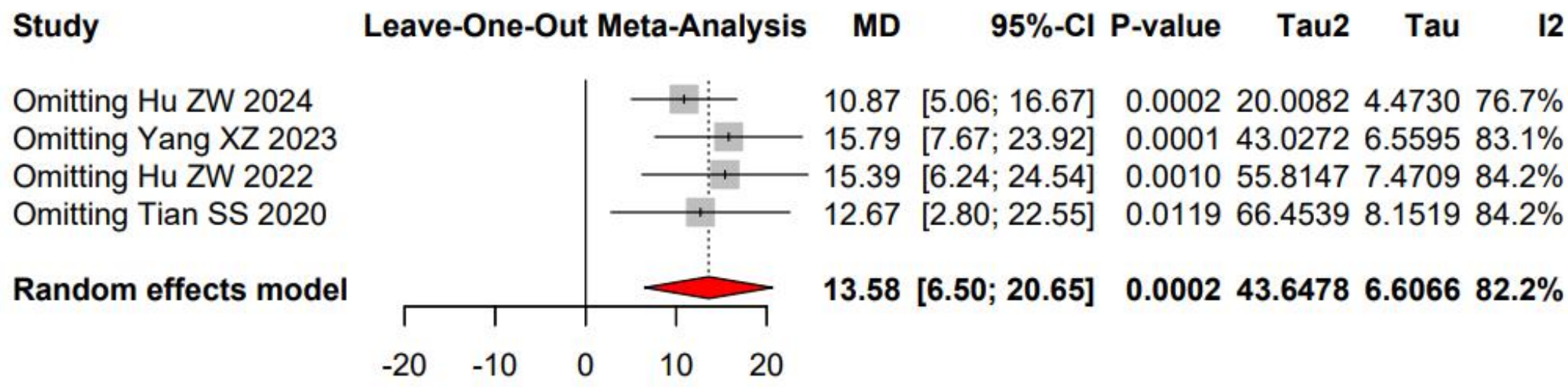

Supplementary Figure S5: Leave-one-out sensitivity analysis for SWAL-QOL.

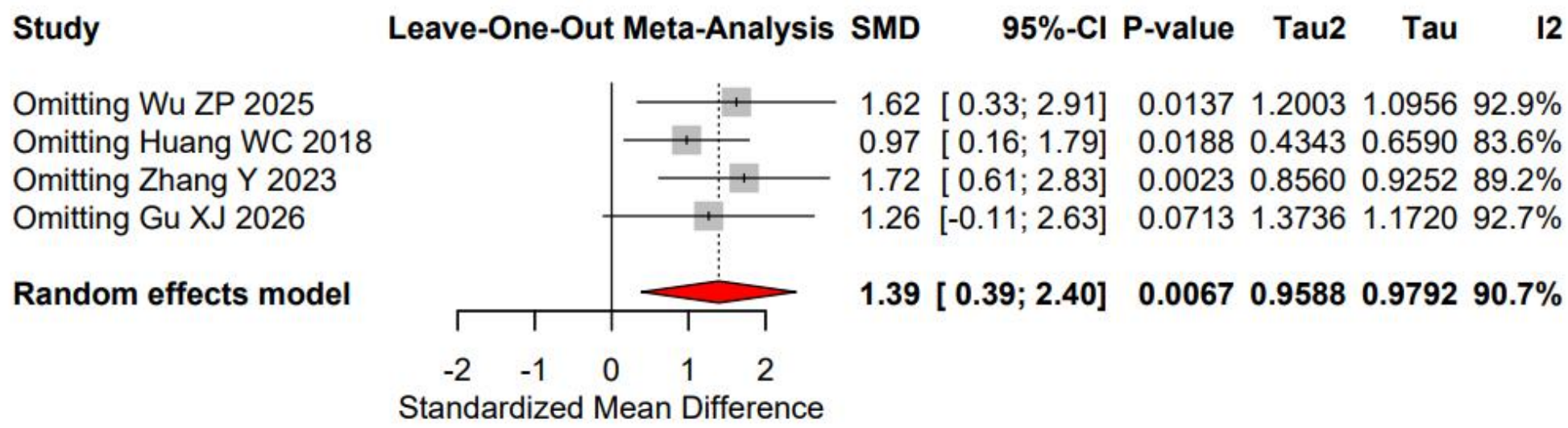

Supplementary Figure S6: Leave-one-out sensitivity analysis for MDADI.

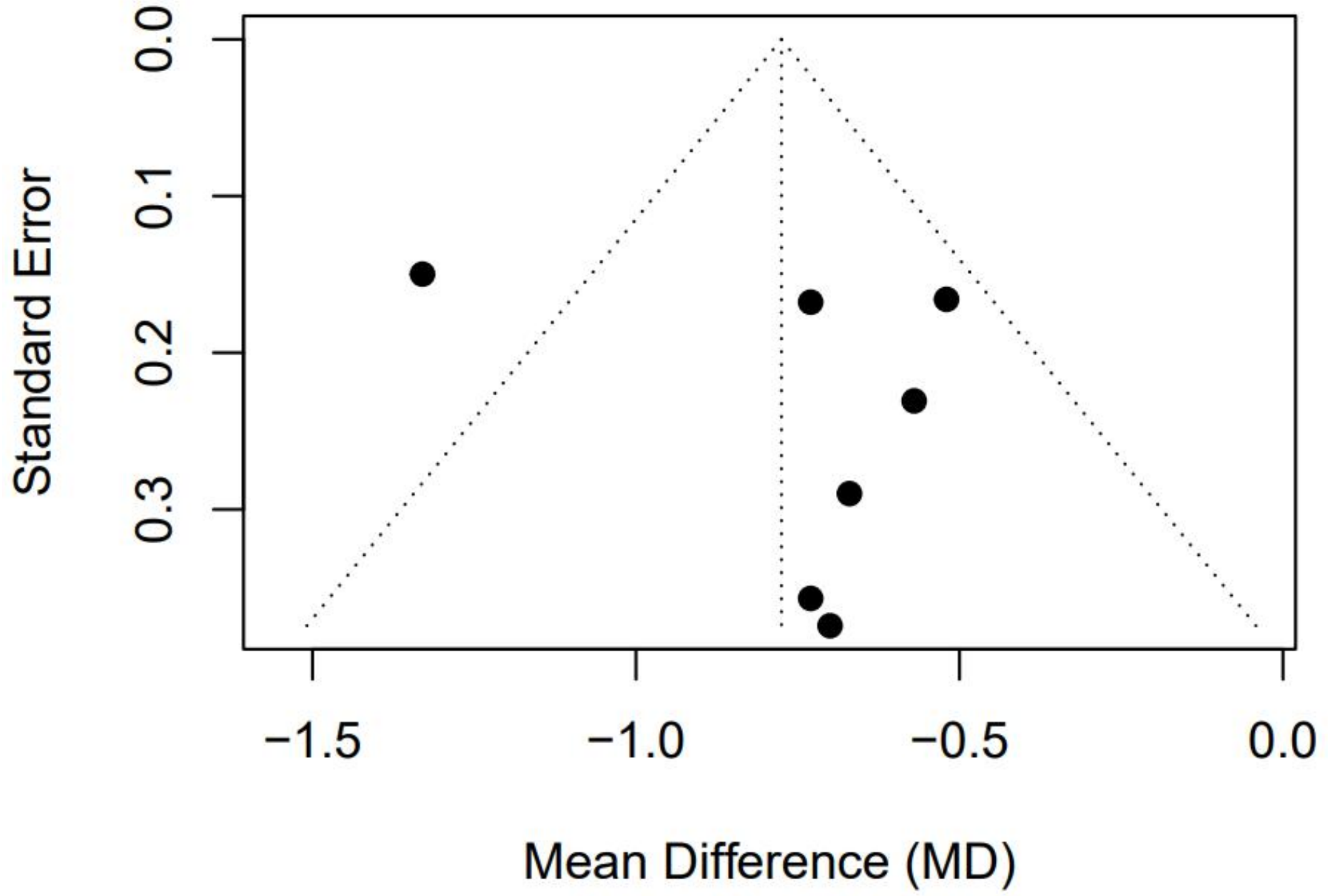

Supplementary Figure S7: A funnel plot of post-treatment WST scores.

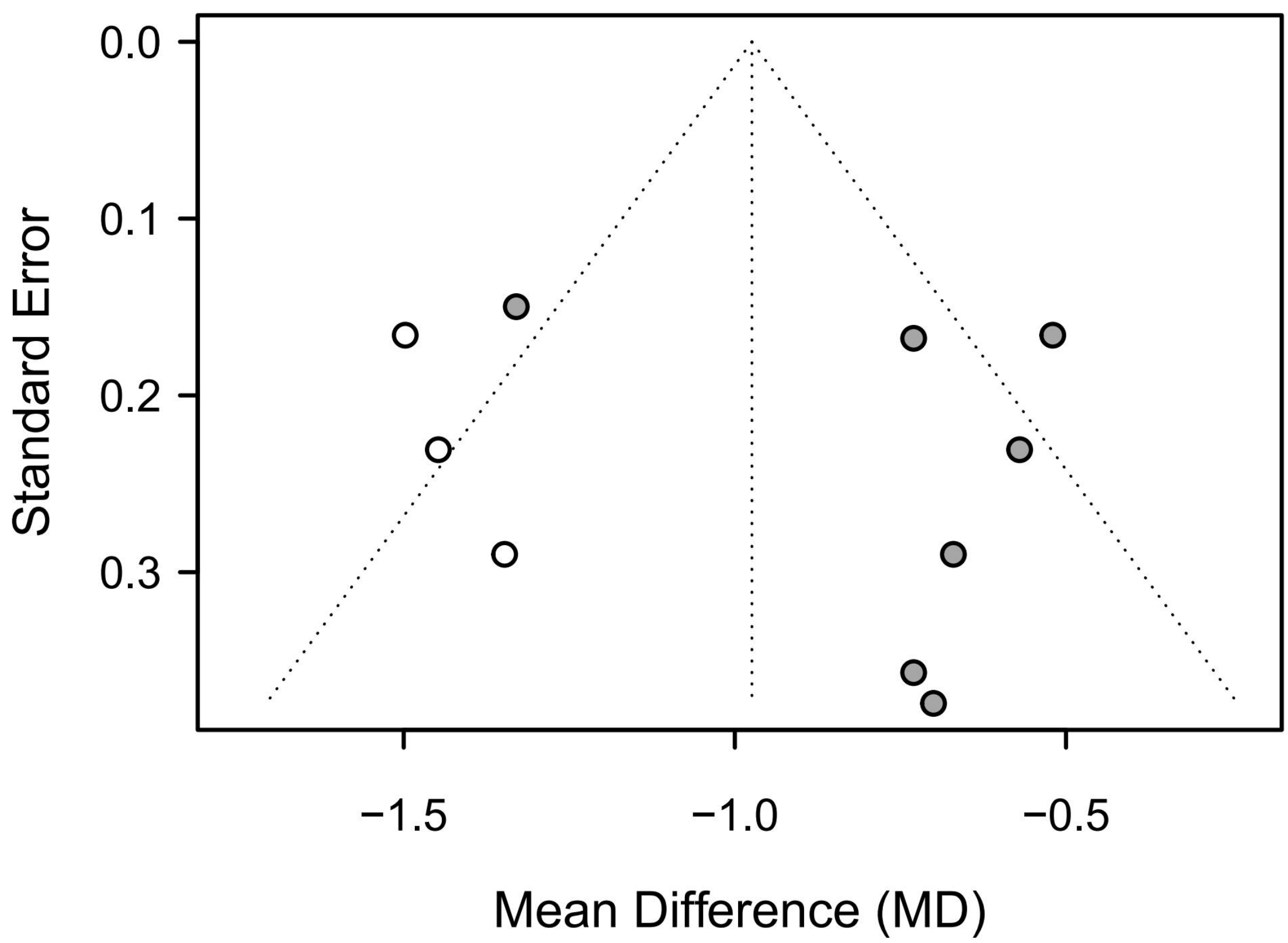

Supplementary Figure S8: Trim-and-fill funnel plot for post-treatment WST scores.

```
Begg's test

Rank Correlation Test for Funnel Plot Asymmetry

Kendall's tau = -0.2381, p = 0.5619


Egger's test

Regression Test for Funnel Plot Asymmetry

Model:    mixed-effects meta-regression model

Predictor: standard error

Test for Funnel Plot Asymmetry: z = 0.6799, p = 0.4965

Limit Estimate (as sei -> 0):  b = -1.0315 (CI: -1.8181, -0.2449)


Note: Exploratory analysis only because the number of studies is limited.
```

Supplementary Figure S9: Begg's and Egger's tests for post-treatment WST scores.
